# Oral Feeding Competences of Healthy Preterm Infants: A Review

**DOI:** 10.1155/2012/896257

**Published:** 2012-05-17

**Authors:** N. Bertoncelli, G. Cuomo, S. Cattani, C. Mazzi, M. Pugliese, E. Coccolini, P. Zagni, B. Mordini, F. Ferrari

**Affiliations:** Department of Pediatrics and Neonatology, Modena University Hospital, 41125 Modena, Italy

## Abstract

*Background.* With increasing sophistication and technology, survival rates hugely improved among preterm infants admitted to the neonatal intensive care unit. Nutrition and feeding remain a challenge and preterm infants are at high risk of encountering oral feeding difficulties. *Objective.* To determine what facts may impact on oral feeding readiness and competence and which kind of interventions should enhance oral feeding performance in preterm infants. *Search Strategy.* MEDILINE database was explored and articles relevant to this topic were collected starting from 2009 up to 2011. *Main Results.* Increasingly robust alertness prior to and during feeding does positively impact the infant's feeding Skills. The review found that oral and non-oral sensorimotor interventions, provided singly or in combination, shortened the transition time to independent oral feeding in preterm infants and that preterm infants who received a combined oral and sensorimotor intervention demonstrated more advanced nutritive sucking, suck-swallow and swallow-respiration coordination than those who received an oral or sensorimotor intervention singly.

The mortality among preterm infants has dramatically decreased in the last decade in the developed countries. Very low-birth-weight (VLBW) infants increased their survival from 50% [1] to more than 85% [2]. However, these VLBW infants are at the greatest risk for medical and developmental sequelae related to their prematurity, which is frequently exacerbated by their prolonged staying in the newborn intensive care unit (NICU) [3–5]. These atypical early experiences alter their development and modify their behaviour [6]. Many clinical factors such as the development of chronic lung disease, recurrent apnoeas and bradycardia, nutritional deficits, as well as stressful environmental conditions, including constant noise, activity, and bright light, may act in combination to impact on the developing preterm infant brain [7]. NICU environment could influence the transition to independent oral feeding and thus delay hospital discharge, negatively affect mother-infant interaction and potentially lead to childhood feeding disorders. As a consequence, there is a need for efficacious early interventions to enhance preterm infants' oral feeding performance [8–10].

The relationship between the maturation of behavioural states, mainly alertness, and the acquisition of oral feeding would deserve investigation [11–16]. The development of this highly complex process reveals a great deal about the maturation of the developing brain and relationships between physiologic and behavioural indices. Among the huge number of routine caregiving interventions in the NICU, it is the successful initiation of oral nipple feeding and attainment of nutritive sucking competence that appears to be the primary determinant of discharge readiness. Attainment of independent oral feeding is one of the criteria recommended by the American Academy of Pediatrics for hospital discharge of preterm infants [17]. The infant's inability to wean from the tube feeding likely will delay hospital discharge and mother-infant reunion and will increase the hospital cost and maternal stress [18, 19]. The sophisticated integration of sucking, swallowing, and breathing with behavioural state is considered the “most highly organized behaviour of the young infant” [20]. There are two dilemmas caregivers face when addressing oral feeding difficulties, that is, infants' ability to complete their feedings safely and the appropriate rate of advancement to independent oral feeding.

McGrath and Medoff-Cooper [21] examined the relationship between the maturation of alertness and the acquisition of nutritive sucking competence during the transition to oral nipple feeding in the NICU. Alertness and nutritive sucking are defined within the context of the synactive theory of development [22, 23]. The synactive theory of development is a model of neurobehavioral development in which the dynamic maturational process of behavioural organization in the preterm infant is illustrated. The integration of the physiologic and behavioural systems is presented within a framework that describes how the maturing infant balances input from the environment while coping with internal physiologic demands. Als et al. [24] have recommended the use of a developmental care approach to promote transition to oral feeding with the reasoning that if an infant's stability, organization, and competence could be enhanced, his/her physiologic and behavioural expression would be optimized. Als proposed that the extent of behavioural organization shown through the infant's unique pattern of behavioural cues and underlying physiologic stability is an indication of central nervous system maturation. Infant behavioural state is starting to be recognized as an important variable in routine caregiving interventions [25]. Alertness is a behavioural state in the normal newborn that is critical to interaction with the environment and has been linked to later cognitive learning and development [26–29]. Typically, caregivers determine oral feeding readiness based on physiologic indicators such as successful weight gain and respiratory stability [30–32]. Postmenstrual age (PMA) or behavioural maturation is often only considered after these other indicators have been satisfied. The infant's ability to reach and maintain robust alertness is seldom used as a meaningful assessment parameter in relationship to oral feeding readiness in the NICU. This study shows that increasingly robust alertness prior to and during feeding does positively impact the infants' feeding competence especially in their ability to generate numbers of sucks and numbers of sucks per burst. Furthermore, the results provide strong support for using the ability to reach and maintain robust alertness as a meaningful readiness parameter for oral nipple feeding.

Oral feeding requires coordination of nutritive sucking, swallowing, and breathing as well.

Oral feeding is not initiated in preterm infants before 32 weeks of PMA mainly because the coordination of sucking, swallowing, and respiration is not established. According to some authors, rhythmic breathing during feeding is first acquired between 34 and 36 weeks' PMA, simultaneously with the maturation of other physiologic processes [33]. In their study, Mizuno and Ueda aimed at establishing normative maturational data for feeding behaviour of preterm infants from 32 to 36 weeks of PMA and evaluating how the relation between swallowing and respiration changed with maturation.

They found that feeding behaviour in preterm infants matured significantly between 33 and 36 of weeks PMA, and swallowing infrequently interrupted respiration during feeding after 35 of weeks PMA. Before 34 weeks' PMA, swallowing occurred usually during a respiratory pause, and after 35 weeks' PMA, swallowing occurred typically at the end of inspiration. They also demonstrated that there was a significant difference in the coordination of swallowing and breathing between <34 and >34 weeks' PMA. The respiratory rate reduction during intermittent sucking after 34 weeks was smaller than that at 32 or 33 weeks' PMA. In preterm infants the maturation in respiration during feeding is not established fully yet at 36 weeks' PMA [34].

Sucking is one of many factors involved in oral feeding [35], and suck-swallow-respiration coordination is a critical factor in achieving safe and successful oral feeding in preterm infants. Sensorimotor intervention is used to improve oral feeding, that is, the provision of developmentally appropriate oral, tactile, kinaesthetic, vestibular, and auditory inputs to facilitate the development of existing skills [36–39]. Oral feeding is a complex multisystem process that involves both the oral and other systems, including cardiorespiratory, gastrointestinal, and neurological [40]. In their study, Fucile et al. aimed at determining whether oral, tactile/kinaesthetic, or combined (oral + tactile/kinaesthetic) interventions enhance oral feeding performance and whether combined interventions have an additive and/or synergistic effect [41]. The authors came to the conclusions that oral and nonoral sensorimotor interventions (i.e. oral and tactile/kinaesthetic) accelerated the transition from introduction of oral feeding to independent oral feeding and enhanced oral feeding skills; sensorimotor interventions had beneficial effects beyond the specific targeted system; combined (oral + tactile/kinaesthetic) sensorimotor intervention had an additive and/or synergistic effect on oral feeding performance over single sensorimotor interventions. Specifically, all three interventions improved proficiency (percent volume taken in first 5 minutes), volume transfer (percent total volume taken), and rate of transfer (mL/min), compared to controls. In conclusion, oral and nonoral sensorimotor interventions provided singly or in combination shortened the transition time to independent oral feeding in preterm infants. These findings demonstrated that sensorimotor interventions have beneficial effects beyond their specific site of input. One limitation of this study was that the three interventions did not reduce the number of days of hospitalization owing to the lack of a specific protocol for discharge planning in the study.

Nutritive suck, suck-swallow and swallow-respiration coordination are key components underlying the improvement of oral feeding outcomes. Knowledge on how the underlying mechanisms mediate these vital coordinative functions is very limited. Fucile et al. [42] investigated the impact of oral and particularly nonoral sensorimotor input (tactile/kinesthetic sensorimotor input to the trunk and limbs) on sucking, swallowing, and respiration. They hypothesized that multiple stimulation sites may potentially impact common underlying systems or may provide multiplicative effects on these coordinative functions. Therefore, the purpose of their study was to further explore whether preterm infants who received an oral and tactile/kinaesthetic, or combined (oral + tactile/kinaesthetic) intervention, before the introduction of oral feeding, demonstrated more advanced nutritive sucking, suck-swallow and swallow-respiration coordination than controls. Preterm infants who received a combined (oral + tactile/kinaesthetic) intervention demonstrated more advanced nutritive sucking, suck-swallow, and swallow-respiration coordination than those who received an oral or tactile/kinaesthetic intervention singly. Results of this study demonstrated that all three interventions impacted to some degree the coordination of the above-mentioned functions related to oral feeding. The oral intervention was the only one that resulted to lead to more mature nutritive sucking skills compared to controls. These improvements may be due to the direct sensorimotor input to the oral musculoskeletal system involved in sucking [43]. It also resulted that duration of the sensorimotor intervention is an important determinant for the achievement of specific nutritive sucking skills.

In their prospective study, Lau and Smith [44] aimed to determine whether the defined oral feeding skills levels can be used as an objective tool for the assessment of preterm infants' oral feeding skills. They hypothesized that the more mature an infant's oral feeding skills level, the better his/her oral performance at that feeding; the more premature an infant, the more immature his/her oral feeding skills level, and the better the oral feeding skills levels, the faster independent oral feeding will be attained. This study demonstrated that the oral feeding skills levels were positively correlated with an infant's feeding performance, that is, the better the levels, the greater the oral performance and the shorter the feeding duration; the oral feeding skills levels positively correlated with gestational age (GA), that is. the less premature the infant, the more mature his/her skills; and the better the skills, the faster the attainment of independent oral feeding. From this study, it is proposed that the use of oral feeding skills levels can offer a more objective indicator of infants' ability to feed by mouth than GA or other tools currently available.

Mention should be made to the debate on whether there is a preferred bottle nipple to be used to enhance the bottle-feeding performance of a preterm infant. scheel et al. hypothesized that feeding performance can be improved by using the bottle nipple with the physical characteristics that enhance infants' sucking skills. A particular bottle nipple that enhanced bottle feeding in healthy VLBW infants was not identified. Based on the notion that afferent sensory feedback may allow infants to adapt to changing conditions, we speculate that infants can modify their sucking skills in order to maintain a rate of milk transfer that is appropriate with the level of suck-swallow-breathe coordination achieved at a particular time [45]. 

Fucile et al. showed that a controlled-flow vacuum-free bottle system versus a standard bottle facilitated overall transfer and rate of milk transfer and shortened oral feeding duration in VLBW infants. Their aim was to understand the basis by which this occurred. They speculated that oral feeding performance improved without significant change in sucking effort with a controlled-flow vacuum-free bottle system as compared to standard bottle. 

In addition, they showed that VLBW infants can tolerate faster milk flow than currently presumed and that the use of a controlled-flow vacuumfree bottle system may reduce energy expenditure as it enhanced feeding performance without increasing sucking effort [46].

##  Conclusions

Inadequate feeding capabilities in preterm infants often lead to poor nutritional and growth failure. Although the prevalence of feeding difficulties in preterm infants is well recognized, the nature of feeding milestones including the timeline of the acquisition of the coordination of sucking, swallowing and respiration still needs more research.

This paper found that increasingly robust alertness prior to and during feeding does positively impact the infant's feeding skills especially in their ability to generate numbers of sucks and numbers of sucks per burst. There is a significant difference in the coordination of swallowing and breathing between <34 and >34 weeks' PMA; in preterm infants the maturation in respiration during feeding is not established fully yet at 36 weeks' PMA. This paper also found that oral and nonoral sensorimotor interventions provided singly or in combination shortened the transition time to independent oral feeding in preterm infants. These findings demonstrated that sensorimotor interventions have beneficial effects beyond their specific site of input. This paper underlined that preterm infants who received a combined oral and sensorimotor intervention demonstrated more advanced nutritive sucking, suck-swallow, and swallow-respiration coordination than those who received an oral or sensorimotor intervention singly. The use of oral feeding skills levels can offer a more objective indicator of infants' ability to feed by mouth than gestational age or other tools currently available. Finally, there were no studies that identified a particular bottle nipple that enhanced bottle feeding in healthy VLBW infants.
